# Remotely sensed above-ground storage tank dataset for object detection and infrastructure assessment

**DOI:** 10.1038/s41597-023-02780-1

**Published:** 2024-01-12

**Authors:** Celine Robinson, Kyle Bradbury, Mark E. Borsuk

**Affiliations:** 1 Department of Civil and Environmental Engineering and Duke Center on Risk, Duke University, Durham, North Carolina 27708 USA; 2 Department of Electrical and Computer Engineering and Nicholas Institute for Energy, Environment & Sustainability, Duke University, Durham, North Carolina 27708 USA

**Keywords:** Energy infrastructure, Civil engineering, Environmental impact, Energy infrastructure

## Abstract

Remotely sensed imagery has increased dramatically in quantity and public availability. However, automated, large-scale analysis of such imagery is hindered by a lack of the annotations necessary to train and test machine learning algorithms. In this study, we address this shortcoming with respect to above-ground storage tanks (ASTs) that are used in a wide variety of industries. We annotated available high-resolution, remotely sensed imagery to develop an original, publicly available multi-class dataset of ASTs. This dataset includes geospatial coordinates, border vertices, diameters, and orthorectified imagery for over 130,000 ASTs from five labeled classes (external floating roof tanks, closed roof tanks, spherical pressure tanks, sedimentation tanks, and water towers) across the contiguous United States. This dataset can be used directly or to train machine learning algorithms for large-scale risk and hazard assessment, production and capacity estimation, and infrastructure evaluation.

## Background & Summary

Many vital industries, including petroleum and chemical production, processing, refining, and transport, rely on short-term storage in above-ground storage tanks (ASTs)^1^. For example, in the United States, the total petroleum storage capacity at sites that commonly use ASTs exceeds two billion barrels^2^. Typical types of ASTs include closed roof, external floating roof, and spherical pressure tanks, the choice of which depends on the characteristics of the stored material, including the specific gravity, volatility, and flash point^3,4^.

ASTs are vulnerable to various hazards, both natural and anthropogenic^5^. For example, during Hurricanes Katrina^6^, Isaac^7^, and Harvey, thousands of barrels of petroleum were released from storage tanks and spread by floodwaters into the environment^8–10^, threatening ecosystems, economies, and human health^11–13^. Internationally, oil depots have been targeted during civil unrest and military conflict, demonstrating the possibility for ASTs to become targets^14,15^. In 2020, as a result of the covid-19 pandemic and the Russia-Saudi Arabia price war, the crude oil market experienced decreased demand and increased inventory, straining storage capacity^16^. Despite these widespread risks, consolidated data suitable for assessing system vulnerabilities and failures, estimating current production and capacity, and evaluating the state of energy and other infrastructure are not readily available to researchers, regulators, and other decision-makers.

The Environmental Protection Agency (EPA) collects information on facilities that process petroleum and hazardous materials through the Facility Registry System (FRS) and Toxic Release Inventory (TRI); however, these datasets do not contain specific tank locations, sizes, or contents^17^. The Emergency Planning and Community Right-to-Know Act (EPCRA) requires that industrial sites make chemical inventory data available to community members via material safety data sheets (MSDS)^18^; however, these data are not available outside of one’s immediate locale.

Remotely sensed imagery has been utilized in some cases to develop AST datasets for specialized purposes. The Oil and Gas Storage Tank (OGST) dataset, for example, identifies tanks located inside the footprint of well pads across Alberta, Canada, in 760 images with resolutions varying from 0.3 to 1.2 meters^19^. The Oil Storage Tank (OST) dataset of external floating roof tanks was developed to estimate capacity and contains 1,595 annotated images taken from Google Earth^20^. A dataset of over 4,500 storage tanks along the Houston Ship Channel (HSC), includes tank location and indication of contaminant barriers, as well as estimates of diameter, height, shell thickness, and content characteristics^21^. Finally, the DOTA dataset includes over 10,000 annotated storage tanks from aerial imagery at various resolutions and locations worldwide^22^. While these datasets all provide useful data in certain contexts, they are generally limited by too few annotations, missing location data, lack of geographic coverage in the United States, simplified classifications, and limited availability.

Recognizing the value of comprehensive, publicly available data for community health and safety risk assessment, petroleum market research, and other analyses, we have developed an original dataset of ASTs from high-resolution aerial imagery across the contiguous United States. It contains geospatial coordinates, border vertices, and orthorectified imagery for over 130,000 spherical pressure, closed roof, external floating roof, sedimentation tanks, and water towers. This dataset was developed for two primary purposes: (1) as training and testing data for subsequent object detection algorithms, and (2) as geospatial data for AST risk assessments. Additionally, the dataset will benefit researchers interested in:

- Petroleum storage working and net capacity estimates;
- Petrochemical market evaluation and economic assessment; and
- Machine learning or computer vision tasks, particularly those that require extensive training datasets.

## Methods

**Fig. 1:**
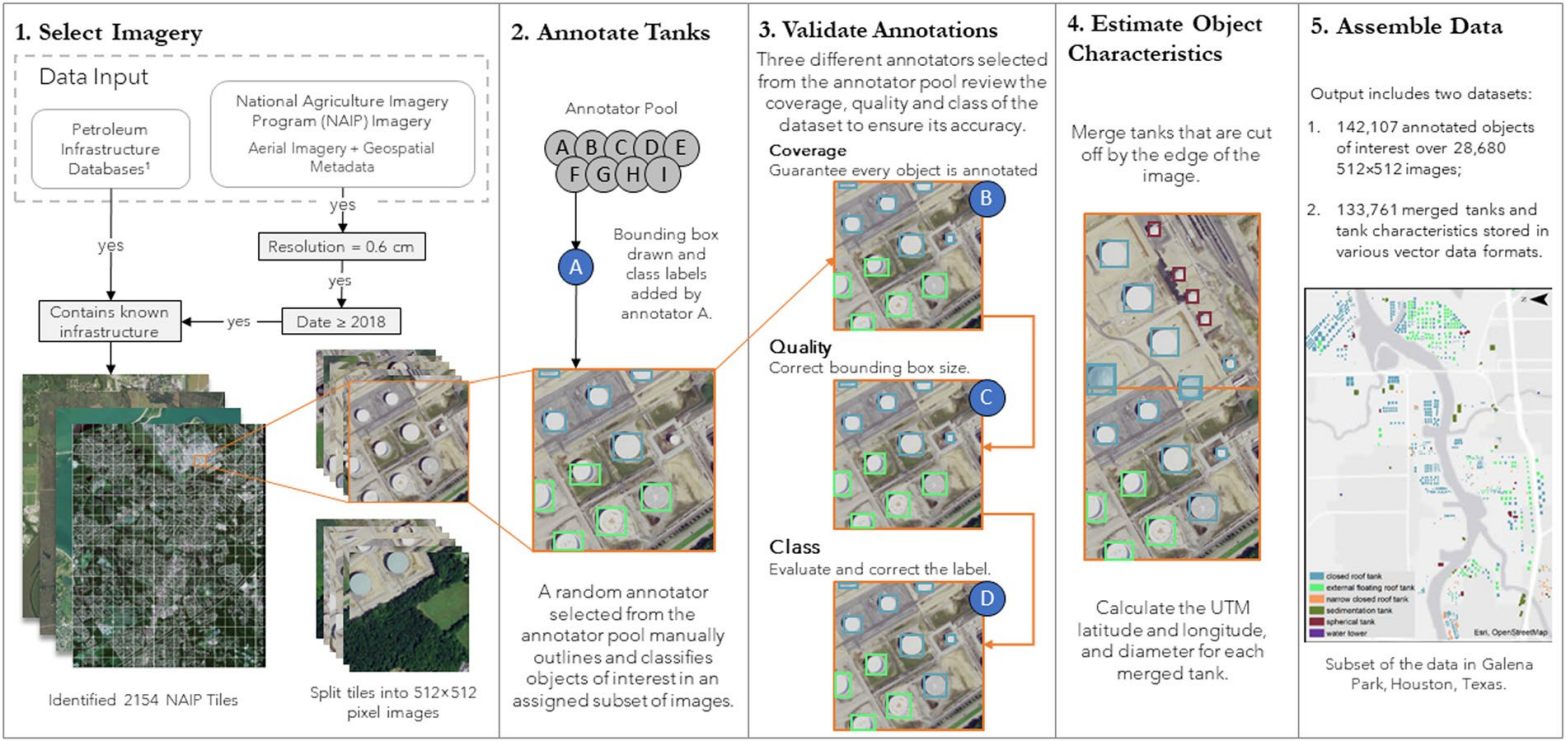
Schematic of the data development and validation process.

We constructed this dataset to contain the imagery, location, bounding boxes, and classifications for various ASTs across the contiguous United States. As illustrated in Fig. 1, our process involved: (1) selecting aerial imagery data, (2) manually annotating and classifying storage tanks, water towers, and sedimentation tanks, (3) validating and correcting when necessary, the bounding boxes and classifications, (4) obtaining geospatial information for each tank, image, and tile, and (5) developing tile level annotations and compiling the full dataset. Each step will be described in the subsections that follow.

### Selecting aerial imagery

The United States Department of Agriculture (USDA) Farm Service Agency’s (FSA) National Agriculture Imagery Program (NAIP) collects high-resolution, remotely-sensed aerial imagery of the continental U.S. during the agricultural growing seasons^23^. We selected NAIP tiles based on two criteria: (1) presence of relevant infrastructure and (2) objects being clearly identifiable. The first criterion was evaluated using existing point datasets of natural gas and petroleum processing plants, reserves, terminals, and ports from the U.S. Department of Homeland Security, Energy Information Administration (EIA), and National Oceanic and Atmospheric Administration (NOAA)^24–30^. As objects of interest range from 3 to 69 meters in diameter, to meet the second criterion only NAIP tiles collected after 2018 were included, guaranteeing a minimum 60 cm ground sampling distance (GSD) so that objects are represented by multiple pixels^23,31^.

**Fig. 2:**
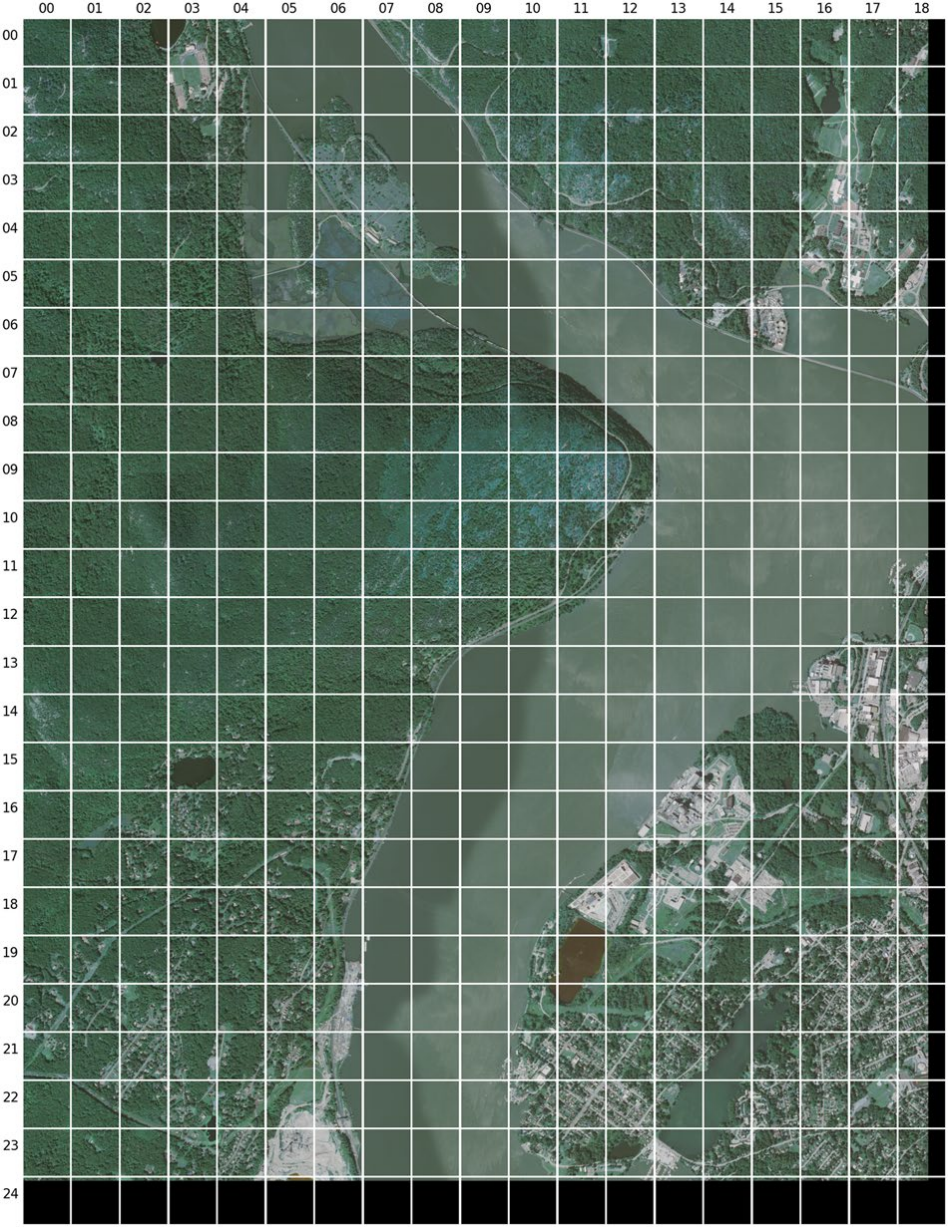
Example of tile (m_4107341_sw_18_060_20190917) split into 512 by 512-pixel images with row and column indices.

A total of 2132 NAIP tiles from 48 states were acquired for annotation from the Microsoft Planetary Computer Data Catalog repository^32^. Each NAIP tile includes four bands (red, green, blue, and near-infrared) over a 3.75-minute longitude by 3.75-minute latitude quarter quadrangle with an additional 300-meter buffer, thereby covering between 12 and 18 square miles^23^. The large size, high-resolution, and high concentration of objects require meticulous annotation^33^. To ensure adequate visual inspection, each NAIP tile was broken into smaller 512-by-512-pixel images, representing approximately 23 acres (0.094 square kilometers) (Fig. 2).

**Fig. 3:**
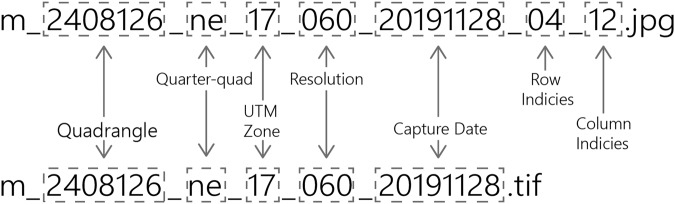
Interpretation of NAIP tile and image file naming convention.

File names for tiles utilize the USDA’s original file naming convention separating the USGS quadrangle identifier corresponding to a 7.5-minute × 7.5-minute area; quarter-quad two-character ordinal direction identifying a given quarter section within a quadrangle; two-digit Universal Transverse Mercator (UTM) zone; image resolution; and capture date in year-month-day, each separated by underscores. 512-by-512-pixel images and image-level annotations utilize the same naming convention and include the row and column index corresponding to the position of the image within the tile (Fig. 3).

### Annotating storage tanks

To accurately and efficiently review and annotate the large number of images identified, we adapted the large-scale annotation procedure developed to create ImageNet^34,35^. Using the NAIP imagery, research assistants manually annotated images using LabelImg, an annotation tool with a graphical user interface (GUI) written in Python^36^. LabelImg allows the researcher to view and identify positive images, or images containing objects of interest, and manually draw rectangular bounding boxes. Annotations and images were further inspected using the zoom in/out function, adjusted using a sidebar with annotation details, or deleted if created in error.

**Fig. 4:**
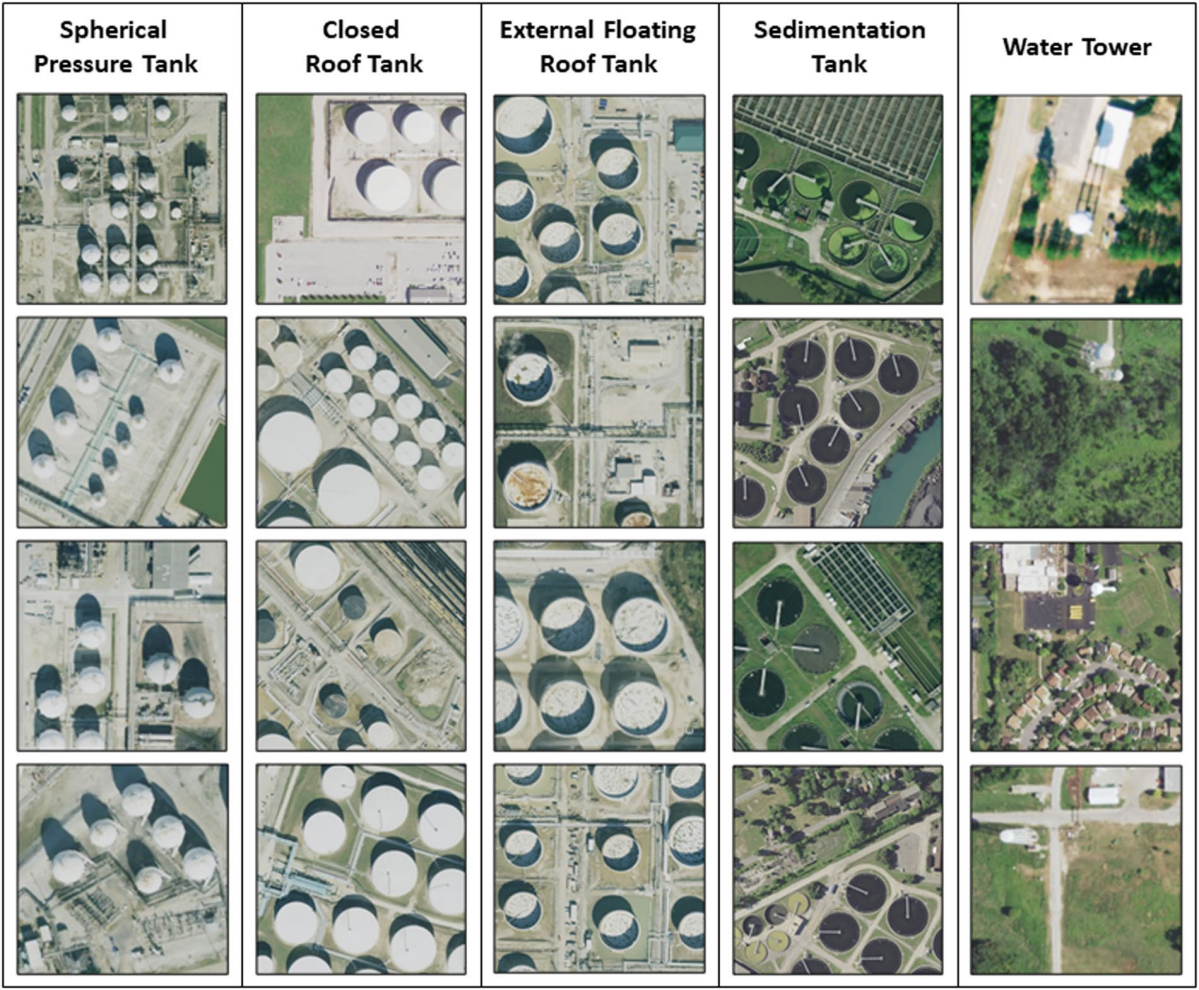
Data samples of each tank type category in the dataset.

Research assistants annotated tanks into one of five categories (Fig. 4). *External floating roof tanks* are cylindrical shells outfitted with a roof that floats on the surface of the stored liquid, rising and falling with the liquid level, and are suitable for liquids with high vapor pressure. By contrast, *closed-roof tanks* have coned, domed, or flat roofs permanently affixed to the tank shell, and generally contain products with lower vapor pressures. Closed roof tanks may also have an internal floating roof. To allow versatility for end users, closed roof tanks less than or equal to 9 meters in diameter are classified as *narrow closed roof tanks*. *Spherical pressure tanks*, also known as Horton spheres, are designed to contain gases or liquids at pressures significantly different than the external environment and to withstand significant internal pressures. Although of less interest for our purposes, *water towers* and *sedimentation tanks* were annotated due to their visual similarity, and in some cases proximity, to ASTs of primary interest. Water towers are tanks elevated to provide pressure for water distribution, while sedimentation tanks are generally used in water and wastewater treatment.

To maintain consistency, research assistants were provided with training on the visual differences between object classes, the LabelImg software, and image annotation protocol. To prevent worker fatigue, research assistants were allocated subsets of the images to annotate on a weekly basis. Due to the low ratio of positive-to-negative images, research assistants reviewed only the positive images and annotations in each subset. The annotation and validation required 22 research assistants to review 22,815 images over 2,300 hours.

### Estimating object characteristics: geographic coordinates and tank diameter

Using the orthorectified NAIP tiles, longitude and latitude coordinates were identified for the northwest and southeast vertices for images and objects. A mesh overlay of the Universal Transverse Mercator (UTM) coordinates was linearly interpolated using the northwest and southeast tile vertices and then transformed into longitude and latitudes using the tile datum. The tile pixel coordinates were then used to obtain each object’s northwest and southwest longitude and latitude coordinates.

Annotated images themselves provide valuable data for a variety of contexts; however, some analyses, particularly infrastructure risk assessment, require data compiled at larger spatial scales and the estimation of additional characteristics, such as location and diameter. Tile level annotations were created to provide this information by translating the image’s pixel coordinates, as well as the row and column position within the tile, to tile pixel coordinates^37^. Approximately 20 percent of the objects in the image level annotations are truncated objects or objects that partially lie outside of the image. Within each tile, truncated objects were merged if they belonged to the same class, separated by a maximum of three meters, and did not intersect. The diameter of each object was calculated as the minimum width or height of each object’s bounding box. Additionally, the capture date is included to allow for longitudinal analyses when multiple tank annotations may exist over time.

### Assembling data

The individual 512 by 512-pixel images and corresponding annotations were compiled into a nationwide dataset along with metadata^37,38^. The annotated dataset includes 142,107 objects distributed across seven classes. Due to the image sampling methods or natural object frequencies, the validated dataset is imbalanced in favour of closed roof and narrow closed roof tanks, which comprise 51% and 35% of the dataset, respectively (Table 1).

**Table 1:** Distribution of objects across classes.

Object Class	Number of Annotations	Percent of Annotations
Closed roof tank	71,990	50.7
Narrow closed roof tank	50,202	35.3
External floating roof tank	10,312	7.3
Sedimentation Tank	5,664	4.0
Water Tower	1,332	0.9
Spherical Tank	1,687	1.2
Undefined Object	904	0.6
Total	142,091	100

**Fig. 5:**
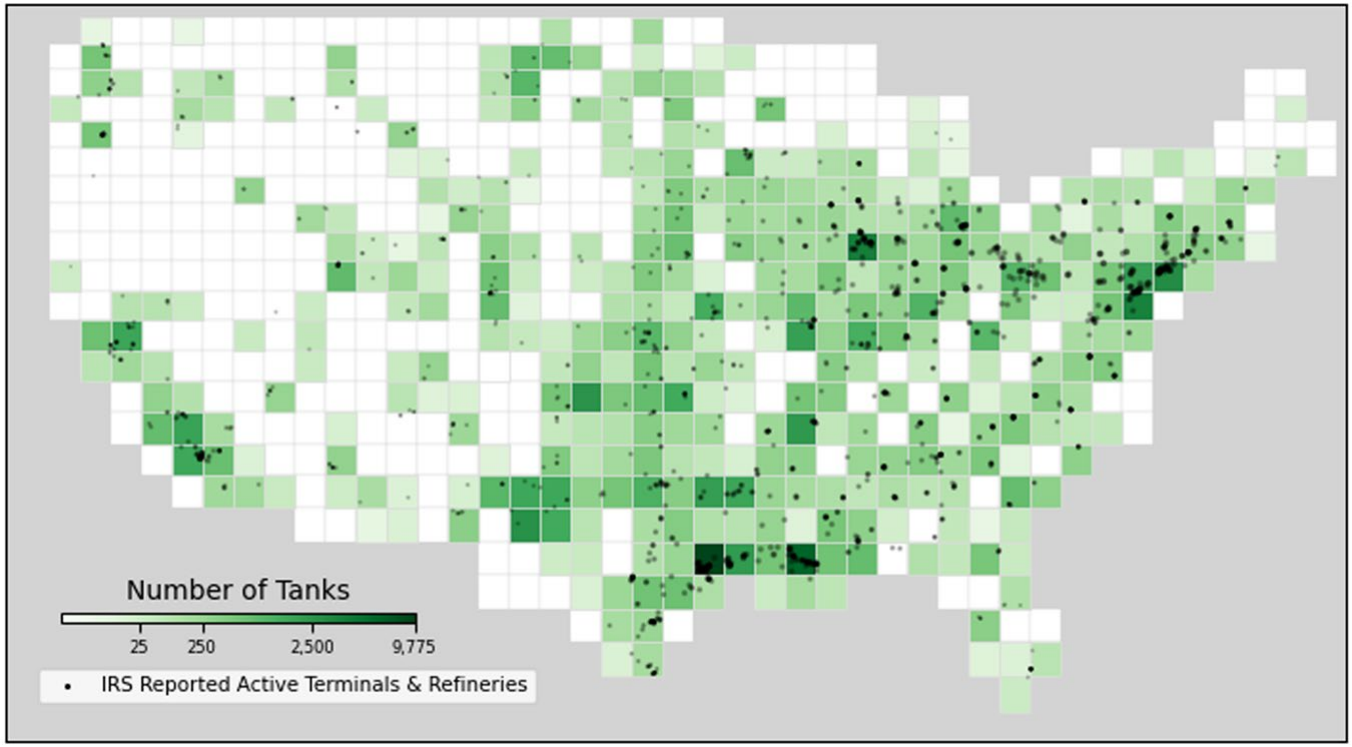
Fishnet grid of the density of annotated tanks in comparison to CONUS and IRS reported active terminals and refineries.

Figure 5 depicts the distribution of annotated objects across the contiguous United States in comparison to active terminals and refineries reported to the Internal Revenue Service^39,40^. This figure illustrates that the spatial coverage of the above-ground storage tank dataset is generally consistent with existing petroleum datasets that were not used to develop our dataset.

## Data Records

Separate data products, including the aerial imagery dataset^38^, above-ground storage tank inventory^37^, and image and tile characteristics^41,42^, totaling 2.3 GB in size, are available in a Figshare repository^43^. Each component is detailed below.

The above-ground storage tank aerial imagery dataset provides a ready-to-use training dataset for deep learning models. This dataset is comprised of two folders which contain 27639 512-by-512-pixel images from 2132 tiles stored in jpg format (“images” folder) and the corresponding annotations in PASCAL Visual Object Classes (VOC) 2007 format as Extensible Markup Language (XML) files (“xmls” folder).

The above-ground storage tank inventory dataset provides cleaned geospatial data and storage tank characteristics for tanks across CONUS. The table is accessible in JSON, GeoJSON, and ESRI shapefile formats to allow for accessibility across applications and software. This dataset is accompanied by a description of the variables included in the inventory dataset.

Metadata relating to the images and tiles are provided by two data records accessible in CSV file format. The image characteristics metadata provides the name of each image and the N.W. and S.E. latitude and longitude coordinates. Additionally, each image can be related to the corresponding parent NAIP tile using the tile name, the image’s row and column index within the tile, and the N.W. and S.E. pixel coordinates respective to the tile. The tile characteristics data record provides the tile name, latitude, and longitude extent of the tile. The image and tile characteristics data records are supplemented with a description of their contents, including the field name, description, data type and units.

## Technical Validation

The development of a high-value, large-scale, multi-class dataset through manual annotation can be laborious and expensive. To efficiently and cost-effectively ensure data quality, we implemented a validation procedure originally developed and described in further detail by Su, 2012^34^. In this validation procedure, we controlled for three potential sources of error: (1) missed objects; (2) poorly drawn bounding boxes; and (3) misclassified objects.

1. *Missed objects*. Relying on human annotators requires that annotators label every object in every image. This is made difficult by potential changes in the appearance of objects due to imagery characteristics such as sensor aperture, cloud characteristics, and time of day when collected.
2. *Poorly drawn bounding boxes*. Rectangular bounding boxes provide the spatial location for an object of interest in an image. When a bounding box is too large, excess pixels are incorrectly noted as part of an object, most commonly occurring with smaller objects. Alternatively, when a bounding box is too small, pixels representing an object of interest are incorrectly excluded (i.e., the legs of water towers or the walls of tanks).
3. *Misclassified objects*. Annotators may confuse: (1) objects of interest for one another, such as external floating roof tanks and sedimentation tanks, or (2) objects of interest with other objects in the image, such as circular buildings, concrete pads, or above-ground pools. In addition, annotators may neglect to indicate when objects are truncated by the edge of the image.

Several procedures were implemented to minimize the effect of these errors and ensure that quality was maintained across all classes and annotators.

### Hiring

Sorokin and Forsyth note that worker compensation may affect the quality of the data annotations, where low wages workers may lose motivation, and high wages would waste resources and attract inefficient workers^44^. Therefore, we controlled the quality of our data set by hiring student research assistants (RAs), allowing our team to carefully screen applicants and employ highly skilled workers who are jointly incentivized by wages and obtained experience.

### Training and feedback

RAs were given extensive training to learn the purpose of the project, use LabelImg, properly annotate images, and understand and distinguish between the object classes. During the training, RAs were instructed to label every object of interest, including those truncated by the edge of an image. To prevent false negatives or mislabeled annotations, annotators were instructed to label ambiguous objects of interest as *undefined objects* so they may be further reviewed during validation. After the completion of the first subset of images and periodically during the annotation phase, RAs were provided with additional training and feedback on their annotations and opportunities to make corrections to minimize the propagation of systematic errors. Additionally, RAs had access to a Slack channel where they could ask for clarification on the annotation procedure, receive feedback on a specific image, and learn from guidance provided to other RAs.

#### Annotation validation

All images and corresponding annotations were reviewed by a set of three RAs using a simple workflow illustrated in Fig. 1. The first RA ensured that every object instance in an image has a corresponding bounding box. This ensures that objects in an image that may have been missed are annotated, particularly small objects, images truncated by the edge of an image, or those outside a cluster of objects. Additionally, they removed bounding boxes drawn in error. The next RA reviewed the quality of each bounding box and adjusted it where necessary to ensure the tightest possible bounding box around every object in each image. The final RA confirmed each annotation was correctly classified, reclassified undefined objects if possible, and checked that truncated objects were correctly marked.

#### Evaluation of validation procedure

**Fig. 6:**
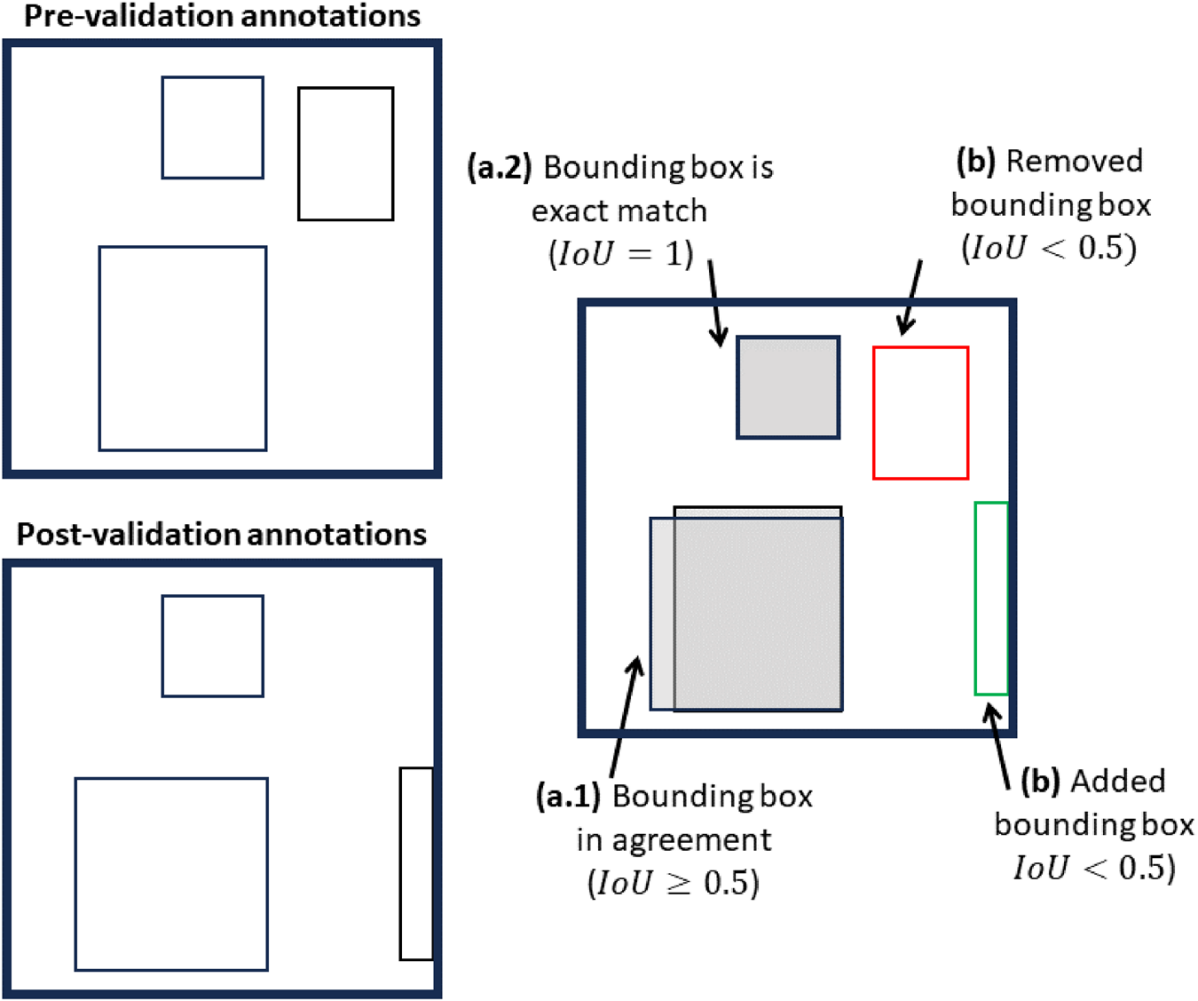
Illustration of the coverage and quality assessment criteria. On the left, pre-validation annotations are shown on top and the post-validation annotations are shown below. Bounding boxes were determined to be in agreement through a multi-step process. For each post-validation bounding box, the intersection-over-union (IoU) was calculated with respect to each of the pre-validation bounding boxes. The highest IoU for a post- and pre-validation bounding box pair was identified. (**a.1**) If the IoU exceeded or equalled 0.5, the post- and pre- validation bounding box pair was determined to be **in agreement**. (**a.2**) Furthermore, if the IoU was 1.0, the bounding box pair was regarded as an **exact match**. (**b**) If the highest IoU for a bounding box was less than 0.5 and the bounding box was present only in the pre-validation or post-validation annotations, we determined that it was **removed** or **added**, respectively, during the validation process.

Since consistency across annotators is a positive characteristic of a dataset, we used multiple metrics to evaluate the changes resulting from the validation procedure. To assess the coverage validation, the number of bounding boxes added and removed during validation was calculated (as illustrated in Fig. 6). The pre- and post-validation datasets contain 128,127 and 142,091 objects, respectively, and it was determined that during the coverage validation process, 0.8 percent (1,067 objects total) of the pre-validation and 10.6 percent (15,032) of the post-validation annotations were removed and added, respectively, as shown in Table 2. To evaluate the quality validation procedure, the number of pre- and post-validation bounding box agreements and exact matches were determined. Of the objects in the post-validation dataset, 89.4 percent (127,050) of the bounding boxes were in agreement and 78.6 percent (111,694) matched exactly with the pre-validation boxes (Table 2). Thus, the difference between bounding boxes in agreement and that exactly match, 10.8 percent (15,356) of the post-validation bounding boxes, required minor adjustments.

**Table 2:** Summary of annotation validation assessment, including the number and relative percentage of the post-validation (i.e., compiled) annotations impacted.

Validation Phase	Assessment	Number of Observations	Percent of post-validation annotations
Coverage	Bounding boxes added	15,032	10.6
	Bounding boxes removed	1,067	0.8
Quality	Bounding boxes in agreement	127,050	89.4
	Bounding boxes with exact matches	111,694	78.6
	Bounding boxes requiring minor adjustment	15,356	10.8
Class	Bounding boxes with object class corrected	8,789	6.2

In analysing the bounding box agreement of the annotations, we did not consider the class labels (tank types). Looking at both the agreement of bounding boxes and the assignment of class labels, 93.8 percent (133,303) are in agreement with the pre-validation bounding boxes and have the same class label, while 73.4 percent (104,344) of bounding boxes exactly match *and* have the same class with respect to the post-validation bounding boxes. To assess the number of changes made to class labels during the class validation phase, the joint frequency of the pre- and post-validation classes for bounding boxes in agreement were tabulated as shown in Table 3. The values on the diagonal of the table indicate the percent of class labels matching pre- and post-validation. The sum of the diagonals, 93.1 percent of the bounding boxes in agreement, indicates that the vast majority of class labels matched before and after validation. The largest share of those that changed class were between closed roof tanks and narrow closed roof tanks. Seven percent of the tanks believed to be closed roof pre-validation were converted to narrowed closed roof tanks, and 5.2 percent were changed vice versa. Given the similarity between these two classes, these changes are not surprising.

**Table 3:** Two-way relative frequency table, expressed as a percent of post-validation objects reporting the object class before and after validation for bounding boxes in agreement.

		Tank type after validation (percent)							
		Closed roof tank	Narrow closed roof tank	External floating roof tank	Sedimentation tank	Water tower	Spherical tank	Undefined object	Total
Tank type before validation (percent)	Closed roof tank	51.20	3.81	0.09	0.03	0.01	0.04	0.02	55.2
	Narrow closed roof tank	1.52	27.43	0.01	<0.01	<0.01	0.01	<0.01	28.97
	External floating roof tank	0.06	<0.01	7.59	0.07	0	0	0.01	7.72
	Sedimentation tank	0.04	0.01	0.11	4.07	<0.01	0	0.02	4.25
	Water tower	0.03	≤0.01	0	<0.01	0.99	0.02	<0.01	1.05
	Spherical tank	0.20	0.02	<0.01	0	0.01	1.21	0.02	1.46
	Undefined object	0.37	0.29	0.04	0.05	0.01	0.01	0.59	1.35
	Total	53.41	31.57	7.84	4.22	1.02	1.28	0.66	100

#### Evaluation of data reliability

Further evaluation of the efficacy of the large-scale validation procedure was conducted on a subset of the data. One percent of the compiled dataset, or 276 images, were randomly selected to be judged according to what we are calling a ground truth dataset. The lead author, with expertise in AST risk assessment, corrected and reviewed each image. To ensure that objects in each image were correctly attributed to the right class, the expert utilized their knowledge, Google Maps, and Google Street View to locate and classify each tank as accurately as possible. This process of checking multiple sources for each tank was far more time-consuming and so is not scalable. However, it provided another assay of the quality of the annotation and validation procedure.

Results from comparing the ground truth data to the results of the validation process revealed an average precision of 0.99 and recall of 0.952 across tank classes. These indicate that the bounding boxes were correctly associated with objects of interest, and fewer than 5 percent of objects were missed. Further inspection offered insight into the causes of missed objects. Of the bounding boxes that were missed, 71.9 percent had a pixel area less than 16^2^ (i.e., an area less than 92.2 square meters) and 93 percent had a pixel area less than 32^2^ (i.e., an area less than 368.64 square meters), indicating that smaller tanks were harder to identify.

## Data Availability

The raw aerial imagery and annotation tools used in this study are publicly accessible^36,45^. The source code developed by the authors to process the imagery and develop the tank inventory dataset are available on GitHub (https://github.com/celinerobi/ast-data-pipeline).
